# Mucinous Cystic Neoplasm of Pancreas in a Pregnant Woman Presenting with Severe Anemia and Gastric Bleeding: Case Report and Review of the Literature

**DOI:** 10.3390/healthcare9050540

**Published:** 2021-05-06

**Authors:** Susan Farahmandi, Mohamed Elessawy, Dirk O. Bauerschlag, Ulrich Pecks, Samir Abdullazade, Jan Henrik Beckmann, Thorsten Heilmann, Anna-Lena Rumpf, Nicolai Maass, Peer Jansen, Vincent Winkler

**Affiliations:** 1 Department of Gynecology and Obstetrics, Campus Kiel, University Medical Center UKSH, Arnold-Heller-Straße 3, Haus C, 24105 Kiel, Germany; Susan.Farahmandi@uksh.de (S.F.); Mohamed.Elessawy@uksh.de (M.E.); Dirk.bauerschlag@uksh.de (D.O.B.); ulrich.pecks@uksh.de (U.P.); thorsten.heilmann@uksh.de (T.H.); anna-lena.rumpf@uksh.de (A.-L.R.); nicolai.maass@uksh.de (N.M.); peer.jansen@uksh.de (P.J.); 2 Department of Pathology, Campus Kiel, University Medical Center UKSH, Arnold-Heller-Straße 3, Haus U33, 24105 Kiel, Germany; Samir.abdullazadeh@uksh.de; 3 Department of General, Abdominal, Thoracic, Transplantation and Pediatric Surgery, Campus Kiel, University Medical Center UKSH, Arnold-Heller-Straße 3, Haus U33, 24105 Kiel, Germany; jan.beckmann@uksh.de

**Keywords:** pregnancy, anemia, mucinous cystic neoplasm, gastric tumor

## Abstract

Mucinous cystic neoplasms of the pancreas are uncommon and especially their occurrence during pregnancy is an extremely rare event which necessitates an individualized and interdisciplinary management. A 33-year old woman was referred to our department during her third trimester of pregnancy (34th week of gestation) with severe anemia and tarry stools. Based on gastroscopic findings, our interdisciplinary team suspected a gastrointestinal stromal tumor and therefore indicated a prompt delivery via cesarean section completed with an oncological resection of the neoplasm. Histological examination subsequently showed a mucinous cystic neoplasm of the pancreas with no evidence of malignancy. To review the prevalence of mucinous cystic neoplasms and to discuss diagnosis and treatment during pregnancy. Moreover, we critically value the indication of preterm delivery and the oncological procedure in the perspective of outcome for mother and infant. A bleeding gastrointestinal tumor during pregnancy represents a life-threatening risk for mother and infant and requires an immediate interdisciplinary treatment. The urgency and radicality of the therapy should be adapted according to individual findings. As our patient’s tumor was suspected of having a malignant progression, an extensive surgical intervention was necessary.

## 1. Introduction

According to the World Health Organization, anemia can be found in about 40% of all pregnancies worldwide. In Germany, providing a well-established health care system with regular prenatal check-ups, the prevalence was reported at a much lower rate, 23%, respectively [1]. In about 75% of all cases the cause of anemia is an insufficient dietary intake of iron or vitamins, both of which can usually be supplemented easily [2,3]. In case of a rapid progression of anemia in pregnancy, other more improbable causes, such as tumors, need to be evaluated. Malignant tumors in pregnancy are a rare complication (1 in 1000 to 1500 pregnancies), but their occurrence has been increasing over recent years [4,5]. The most common malignant tumor in pregnancy is breast cancer (1 in 3000–10,000 pregnancies) followed by cervical cancer (0.5 in 10,000 pregnancies) [6,7]. Other even more rare malignancies in pregnancy include ovarian cancer, melanomas and hematologic or intestinal malignancies.

Rarely, a benign neoplasia can also be the cause of anemia. One of these rare tumor entities is the mucinous cystic neoplasm (MCN) of the pancreas. Especially its occurrence in pregnant women represents a rare event [8]. The majority of MCNs occur in women (female-to-male ratio is 20:1) and the mean age at time of diagnosis is between 40 and 50 years [9,10,11]. Usually it is located in the pancreatic body or tail (95–98%) [12]. Those mucin-producing neoplasms rarely cause any specific symptoms and therefore generally are incidental findings during scanning for other indications or routine check-ups [11,13]. Some patients show unspecific symptoms such as: mild abdominal pain, nausea and vomiting, back pain, epigastric heaviness and fullness [9,11,14,15]. Less frequently, complications like pancreatitis, jaundice [8,14], and bleedings or anemia may occur [16].

**Figure 1:**
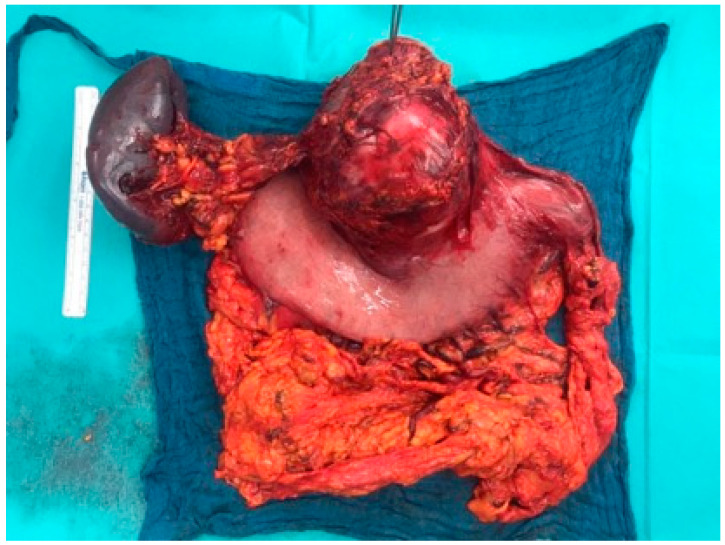
Macroscopic overview of the tumor from dorsal side. The tweezer marks the pancreatic resection margin with the passing lienal artery.

Histologically, MCNs consist of two definite components: a mucin-producing columnar epithelial layer and a unique ovarian-type subepithelial stroma [11], which cannot be found in any other pancreatic neoplasm [17] (Figure 1.) The underlying ovarian-type stroma is defined by densely arranged spindle-shaped cells with oval nuclei and sparse cytoplasm [17]. The origin of this stroma in MCNs remains unclear, but its histological presence is mandatory for diagnosis [11,18]. The mucin-producing epithelial layer appears with different grades of dysplasia. The current two-tiered grading system for MCN recently replaced the former three-tiered grading scheme; neoplasms belonging to the former categories of “MCN with low-grade dysplasia” and “MCN with intermediate-grade dysplasia” are now categorized as low-grade MCN, and those belonging to the former category of “MCN with high-grade dysplasia” are now categorized as high-grade MCN [19]. Therefore, MCNs are considered precursors to invasive pancreatic cancer, typically tubular adenocarcinomas [12]. The incidence of invasive carcinomas in MCNs varies from 6% to 36% [12].

## 2. Case Presentation

We present a primigravid, 33-year old European woman, who was admitted to the emergency department for obstetrics in her 34th week of pregnancy, due to a symptomatic but compensated anemia, which clinically has been manifesting in a 3-week history of fatigue, mild circulatory insufficiency and a hemoglobin level of 4.8 g/dL. The patient negated vaginal or rectal bleedings but mentioned a blackening of stool during the last three weeks, which she attributed to the intake of iron supplements. There were no abnormal findings considering the pregnancy. After transfusion of three packed red blood cells, only a marginal increase in hemoglobin level (5.9 g/dL) was recorded. Considering the symptoms and insufficient increase in hemoglobin levels, a gastrointestinal bleeding was assumed and gastrointestinal endoscopy was advised. Gastroscopy revealed a posterior wall-sided mobile conglomerate tumor in the corpus and antrum with an ulcerated and hypervascularized surface leading to severe hemorrhage. Proximal to the tumor, the posterior wall of the gastric corpus was impressed extraluminally. According to its endoscopic appearance, the tumorous mass was assumed as a highly suspicious finding with the suggestion of an ulcerated malignant gastric tumor or gastrointestinal stromal tumor (GIST) of the stomach. Biopsy or intervention measures were not performed due to the risk of further uncontrollable bleeding. The extent of the tumor was additionally determined by transabdominal ultrasound, which showed an 11 cm mostly cystic tumor mass presumably arising from the pancreatic tissue. At this point, the entity was still not clear. An interdisciplinary team of gynecologists, visceral surgeons and gastroenterologists evaluated these clinical findings with regard to the gestational age and decided to refrain from further diagnostics (magnetic resonance imaging (MRI)/computed tomography (CT)/biopsy/blood tests) and conservative therapy because of the urgent need of maternal treatment. A preterm delivery via cesarean section was indicated and followed by visceral surgery in the same session. Intraoperatively, a differentiation between a conglomerate tumor arising from the gastric posterior wall or the pancreas was impossible (Figure 1). A tumor resection was performed, while preserving a small gastric pouch and the post-pyloric duodenum. A distal pancreatectomy and, as the splenic vein seemed to be infiltrated as well, a splenectomy with resection of the splenic vessels followed. Finally, a reconstruction of the gastrointestinal tract analogical to commonly performed bariatric surgeries, speaking of a Roux-en-Y reconstruction with gastrojejunostomy, completed the surgery. Macroscopically, there were no signs of intraabdominal metastases. The postoperative recovery remained without complications. The premature eutrophic infant (1900 g, APGAR 7/8/9, pH 7.36) only had a short adaption disorder and CPAP (continuous positive airway pressure) therapy in the first minutes of life. There were no further major events in the treatment of the newborn.

**Figure 2:**
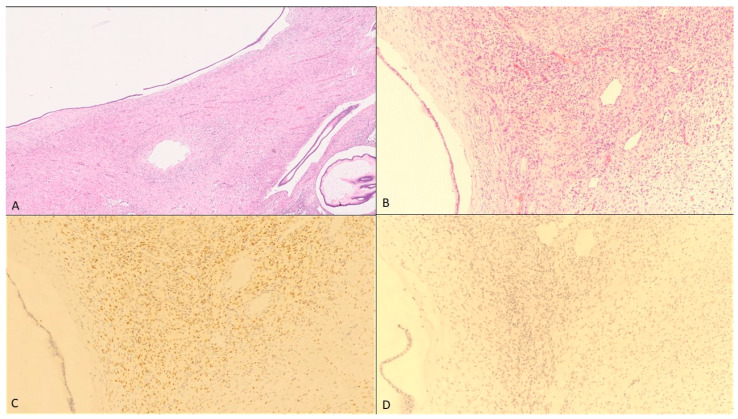
Pathological findings. (**A**) Cyst lined by mucinous epithelium (hematoxylin-eosin, 40×), (**B**) Ovarian-type stroma consists of spindle-shaped cells with round or elongated nuclei and sparse cytoplasm (hematoxylin-eosin, 200×), (**C**) Immunohistochemical staining with the expression of progesterone receptor (200×) and (**D**) with the negativity for estrogen receptor in the ovarian-type stroma (200×).

Pathology confirmed a 12.5 cm × 11 cm × 10.5 cm measuring, mostly cystic, partly necrotic, knotty pre-bulged tumor, which was located on the posterior gastric wall and was removed with tumor-free resection margins (Figure 1). The spleen and twenty-five lymph nodes were not affected. Multi-chambered cystic structures, lined by a mucin-producing columnar epithelial layer and ovarian-type stroma consisting of spindle-shaped cells with round or elongated nuclei and sparse cytoplasm, were observed through light microscopy (Figure 2A,B). Further immunohistochemical staining confirmed the expression of progesterone receptors (PR) and a negativity for estrogen receptors (ER) in the ovarian-type stroma (Figure 2C,D). Thus, unexpectedly, the final diagnosis of a pancreatic mucinous cystic neoplasm (MCN) with low grade dysplasia was made. More specifically, no evidence of malignancy was found.

## 3. Discussion

Pancreatic mucinous cystic neoplasms represent highly rare findings. Especially their occurrence in pregnant women has only been published in a few reports (Table A1). MCNs usually grow slowly and are mostly benign, as they do not infiltrate surrounding tissues. Nonetheless, most case reports have mentioned considerably rapid growth and larger size during pregnancy, which may also increase the risk for malignant transformation into invasive carcinomas, compressing of surrounding tissues, pancreatitis and potentially leading to tumor rupture or fetal hazards including intrauterine growth restriction (IUGR) [20,21,22,23].

The present case showed exceptional displacing tumor growth causing a compression of the posterior gastric wall with consecutive erosion of the mucosa and severe gastric bleeding.

MCNs in pregnant women have been described to exhibit a greater size (14.5 cm) if compared to MCNs in the non-pregnant population (average age 40–45 years, 84% < 60 years) (6.5 cm) [24,25]. This might be caused by the expression of estrogen-receptors (ER) and progesterone-receptors (PgR) in the ovarian-type stroma of pancreatic MCNs, following the hypothesis that female sex hormones greatly influence their biological behavior [17,21,22,23,26]. Previous case reports confirmed the existence of ER (30% of cases) and PgR (60–90%) in the ovarian-like stroma in pregnant women as well as in non-pregnant women [12] (Table A1). Nevertheless, it remains unclear whether high levels of female sex hormones during pregnancy directly stimulate the transformation of MCN into malignant tumors [15,27,28]. Within the 35 case reports of MCNs occurring during pregnancy, we have found nine cases (25.7%) with an invasive carcinoma (Appendix Table A1), whereas the malignancy rate in the non-pregnant population varies between 3.9% and 16.3% [12]. Interestingly, the absence of progesterone receptors correlated with worse outcomes according to Thompson et al. and Robles-Diaz et al. [15,29]. Progesterone might suppress the malignant transformation of epithelial cells in the ovarian-like stroma of MCNs. Immunohistochemical staining in our patient’s tumor tissue also revealed a positivity for progesterone-receptors (Figure 2C) and therefore received growth impulses but also suppressing signals preventing malignant transformation. In a non-critical situation with a clinically stable patient, the diagnostic and treatment of MCNs usually requires a carefully conducted preoperative assessment which includes the evaluation of clinical features and imaging techniques like transabdominal ultrasound, MRI, CT and eventually endoscopic ultrasound sonography (EUS) with fine needle aspiration (FNA). In most cases, urgent interventions are not needed [12], but in our case the diagnostic and treatment modalities were limited by pregnancy and the urgency of the clinical presentation. Neither an MRI, nor an EUS-FNA would have impacted the clinical management of our patient and would have only delayed the urgently needed surgical intervention. According to the European evidence-based guidelines on the management of pancreatic cystic neoplasms (2018) an endoscopic ultrasound sonography with fine needle aspiration is not indicated if a definitive indication for surgery exists [30]. Additionally, the preoperative grading of mucinous neoplasms in biopsies remains a challenge and leaves an uncertainty due to limited sampling, since their grades of dysplasia often vary within the tumor [16]. As a consequence, practitioners should strive for minimizing both the maternal and fetal risk and therefore perform further diagnostics in a suitable clinical situation. To date, the management of pancreatic cystic neoplasms still has not been standardized and the decision on observing/conservative or active management is based on expert opinions and only a few case reports. Particularly their occurrence during pregnancy is associated with more factors (i.e., gestational age, fetal-maternal impairment) that have to be considered in the decision making. In our case, we decided to perform a surgical excision of the tumorous mass in a standard oncological procedure even though its exact origin and invasion was unclear. According to the European evidence-based guidelines (2018) all MCNs *≥* 40mm should undergo a surgical resection, as well as MCNs which are symptomatic or show risk factors for malignant transformation, regardless of their overall size (Grade 1B, strong agreement) [13,23,26,30]. The incidence of malignant transformation in MCNs correlates directly with their size, their growth rate and their cysts’ complexity [13]. In order to avoid incomplete surgical treatment of MCNs with associated invasive carcinoma, a standard oncological resection (distal pancreatectomy in 90–95% of MCN cases) with lymph node dissection and splenectomy is indicated for all patients whose preoperative imaging indicates high grade dysplasia or association with an invasive carcinoma (Grade 1B, strong agreement) [30]. MCNs without risk factors and a very low probability of malignancy can be treated in a non-oncological manner (distal pancreatectomy with splenic preservation or even parenchyma-sparing pancreatectomy) [30]. In hindsight, a non-oncological surgery also would have been possible for our patient if a malignancy could have been excluded for sure. Therefore, further diagnostics would have been necessary, for which the patient needs to be clinically stable. In our case however, there was on the one hand an active bleeding which needed urgent surgery on the other hand we expected a malignant process. This led to the decision of a prompt surgery. In the 34th week of pregnancy the risk for the infant was also justifiable, even without respiratory distress syndrome (RDS) prophylaxis. Following complete surgical resection, patients with MCN show a 5-year survival rate of 100%, whereas MCN patients with an associated invasive carcinoma have a 5-year survival rate of only 20% to 75% depending on resection status, tumor size and grading [12]. A follow-up, after a complete resection of MCNs without associated invasive carcinoma, is usually not needed, since numerous studies have shown that the risk of tumor recurrence is nil and the overall survival rate is 100% [11].

## 4. Conclusions

Pancreatic mucinous cystic neoplasms are a rarity and their occurrence in pregnant women requires careful interdisciplinary management. The rapid tumor growth during pregnancy is associated with an increased risk for malignant transformation and associated complications like tumor rupture, pancreatitis, fetal intrauterine growth restriction or in this case severe anemia. The risk of disease progression in pregnancy should be weighed against an observative strategy in order to ensure fetal maturity. Close cooperation between different medical disciplines (gynecologists, neonatologists, visceral surgeons and pathologists) is vital in order to properly manage MCNs in pregnant women.

## Data Availability

The data presented in this study are available on request from the corresponding author. Otherwise see the Table A1. “Summary of previously reported pancreatic MCNs associated with pregnancy”.

## Appendix A

**Table A1:** Summary of previously reported pancreatic MCNs associated with pregnancy.

No.	Author/Citation	Age at Diagnosis	Gestational Age at Diagnosis (Weeks)	Maximum Diameter of Tumor (cm)	Location in Pancreas	Timing of Operation	Complications	Surgical Procedure	Histological Diagnosis	ER/PR
1	Smithers [31]	33	7	10	Body/tail	AD (abortion in week 9)	Tumor rupture	DP	adenocarcinoma	NA/NA
2	Baiocchu [32]	29	40	10	Tail	AD (NA)	No	DP	adenocarcinoma	NA/NA
3	Olsen [33]	25	5	5	Tail	DPr (week 18)	NR	DP	Adenoma	NA/NA
4	Ganepola [34]	37	4	12	Tail	DPr (week 23)	NR	DP	Adenoma	+/+
5	Kato [21]	33	15	22	Body/tail	DPr (week 23)	IUGR	DP	Adenoma	+/+
6	Lopez-Tomassetti Fernández [23]	26	20	15	Tail	DPr (week 26)	Episodic epigastric pain	DP	Adenoma	NA/NA
7	Kitagawa [35]	25	AD	15	Body	AD (11 months)	No	DP	Adenoma	−/+
8	Herring [36]	34	3	20	Body/tail	DPr (week 17)	NR	DP	adenocarcinoma	+/+
9	Ozden [37]	32	36	15	tail	AD (emergency Caesarean: week 34)	Tumor rupture	DP	adenocarcinoma	−/−
10	Berindoague [38]	31	AD	12	Body/tail	AD (2 months)	NR	DP	adenocarcinoma	−/−
11	Ishikawa [39]	33	17	18	Body/tail	AD (3 months)	NR	DP	Adenoma	−/−
12	Ikuta [40]	30	10	18	Tail	AD (abortion: week 10)	Missed abortion	DP	Moderate dysplasia	+/+
13	Hakamada [41]	38	2 years before pregnancy	14	Tail	DPr (second trimester)	nausea, hematemesis, tarry stool	DP, partial stomach resection	Borderline	NA/+
14	Wiseman [42]	32	11	15	Tail	DPr (week 15)	Intractable nausea	DP	Low grade dysplasia	+/+
15	Brown [16]	38	8	10	Body/tail	DPr (week 8)	Gastrointestinal bleeding	DP	Severe dysplasia	NA/NA
16	Shirakawa [43]	34	26	19	Body/tail	AD (3 months)	No	DP	Adenoma	+/+
17	Shirakawa [43]	36	AD	16	Body/tail	AD (NA)	No	DP	adenocarcinoma	−/−
18	Asciutti [44]	31	23	8	Tail	AD (1 month)	Pancreatitis	DP	Adenoma	NA/NA
19	Coral [45]	26	3	32	Body/tail	AD (1 month)	No	DP	Adenoma	−/+
20	Naganuma [22]	32	33	11	Head	AD (emergency Caesarean: week 34)	Tumor rupture	PD	adenocarcinoma	−/+
21	Martins-Filho [46]	20	20	15	Body/tail	DPr (week 20)	No	DP	Adenoma	NA/NA
22	Boyd [47]	21	7 months before pregnancy	17	Body/tail	DPr (week 20)	Abdominal distension and fullness	DP	Moderate dysplasia	NA/NA
23	Iusco [48]	28	AD	16	Body/tail	AD (NA)	No	DP	Associated adenocarcinoma	+/+
24	Tsuda [49]	28	Some years before	15	Body/tail	DPr (week 18)	No	DP	Severe dysplasia	+/+
25	Tica [8]	27	29	15	Body/tail	AD (2 months)	NR	DP	Adenoma	−/−
26	Urabe [50]	34	16	16.5	Body	AD (1 month)	No	DP	Adenoma	NA/NA
27	Urabe [50]	40	33	12	Tail	AD (emergency Caesarean: week 33)	Rupture	DP	Adenoma	+/+
28	Takashima [51]	28	12	13	Head	AD (1 month)	No	Enucleation	High grade dysplasia	+/+
29	Kleeff [52]	41	Three years before pregnancy	7	Body/tail	AD (3 months)	No	DP	Moderate dysplasia	+/+
30	Kosumi [26]	33	4	7.6	Body/tail	AD (2 weeks)	No	DP	NA	+/+
31	Veits [53]	28	11	4.7	Tail	DPr	Pancreatitis	DP	Low grade dysplasia	NA/NA
32	Revoredo [12]	38	17	20	Body/tail	DPr (week 29)	Rupture	DP	Moderate dysplasia	−/+
33	Revoredo [12]	30	18	13.6	Body/tail	DPr (week 20)	no	DP	adenocarcinoma	+/+
34	Carvalho [13]	32	31	24	Tail	AD (1 month)	no	DP	Low grade dysplasia	NA/NA
35	Present case	33	34	12.5	Body/tail	AD (emergency Caesarean: week 34)	Gastrointestinal bleeding	DP + 4/5-gastrectomy	Low grade dysplasia	−/+

AD—after devlivery; DPr—during pregnancy; PD—pancreatoduodenectomy; DP—Distal pancreatectomy; NA—not available, NR—not reported.
